# The Importance of Steroid Uptake and Intracrine Action in Endometrial and Ovarian Cancers

**DOI:** 10.3389/fphar.2017.00346

**Published:** 2017-06-19

**Authors:** Tea Lanišnik Rižner, Theresia Thalhammer, Csilla Özvegy-Laczka

**Affiliations:** ^1^Institute of Biochemistry, Faculty of Medicine, University of LjubljanaLjubljana, Slovenia; ^2^Department of Pathophysiology and Allergy Research, Centre for Pathophysiology, Infectiology and Immunology, Medical University of ViennaVienna, Austria; ^3^Momentum Membrane Protein Research Group, Research Centre for Natural Sciences, Institute of Enzymology, Hungarian Academy of SciencesBudapest, Hungary

**Keywords:** sulfatase, aromatase, 17beta-hydroxysteroid dehydrogenase, transporters, OATP, ABC-transporter

## Abstract

Endometrial and ovarian cancers predominately affect women after menopause, and are more frequently observed in developed countries. These are considered to be hormone-dependent cancers, as steroid hormones, and estrogens in particular, have roles in their onset and progression. After the production of estrogens in the ovary has ceased, estrogen synthesis occurs in peripheral tissues. This depends on the cellular uptake of estrone-sulfate and dehydroepiandrosterone-sulfate, as the most important steroid precursors in the plasma of postmenopausal women. The uptake through transporter proteins, such as those of the organic anion-transporting polypeptide (OATP) and organic anion-transporter (OAT) families, is followed by the synthesis and action of estradiol E2. Here, we provide an overview of the current understanding of this intracrine action of steroid hormones, which depends on the availability of the steroid precursors and transmembrane transporters for precursor uptake, along with the enzymes for the synthesis of E2. The data is also provided relating to the selected transmembrane transporters from the OATP, OAT, SLC51, and ABC-transporter families, and the enzymes involved in the E2-generating pathways in cancers of the endometrium and ovary. Finally, we discuss these transporters and enzymes as potential drug targets.

## Hormone-dependent cancers

Steroid hormones have important roles in human physiology, and the disruption of androgen, estrogen, and progesterone actions are implicated in the development of hormone-dependent cancers and benign hormone-dependent diseases. Hormone-dependent cancers include prostate, breast, endometrial, and ovarian cancers, which comprise more than 20% of all cancers in humans, and more than 35% of cancers in women (Ferlay et al., 2013). Indeed, in women, breast cancer is the most frequent cancer in the developed world. In 2012, 1,671,149 new cases of breast cancer were estimated worldwide, with 521,907 associated deaths. In the developed world, endometrial cancer is the most common among gynecological cancers. Worldwide, there were 319,605 new cases and 76,160 deaths from endometrial cancer in 2012 alone (Ferlay et al., 2013). The most deadly of the hormone-dependent cancers is ovarian cancer. Worldwide, there were 238,719 new cases and 151,905 deaths from ovarian cancer in 2012 (Ferlay et al., 2013).

Cancers of the endometrium and ovary predominately affect women after menopause, and they are more frequently observed in developed countries. These enormous numbers of patients and deaths attributed to these hormone-dependent diseases demonstrate the uttermost importance of detailed understanding of their pathophysiology, including the roles of the steroid hormones.

### Endometrial cancer and steroid hormones

Endometrial cancer is the fourth most prevalent among all cancers, and the eighth most deadly cancer in North American and European women (Ferlay et al., 2013). The incidence of endometrial cancer is also increasing, with more patients from the premenopausal and peri-menopausal population (Sanderson et al., 2017). Based on its histopathology, clinical manifestation, and epidemiology, endometrial cancer cases can be classified into two groups. Estrogen-dependent type I endometrial cancer with endometrioid or mucinous histology includes 70–80% of all cases. Type II endometrial cancer is characterized by serous papillary or clear-cell histology, originates from atrophic endometrium, and develops from intraepithelial carcinoma (Amant et al., 2005; Ryan et al., 2005). Type II endometrial cancer includes 20% of all cases, and it is usually considered as estrogen independent.

Endometrial cancers also differ in their genetic alterations. Type I tumors are commonly associated with microsatellite instability and mutations in the *PTEN, PIK3CA, K-RAS, CTNNB1*, and *FGFR* genes (Yeramian et al., 2013). Type II endometrial cancers are associated with inactivation of the *TP53* and *TP16* genes, and with amplification of the *ERBB, CCND1*, and *CCNE1* genes (Yeramian et al., 2013; Murali et al., 2014). Based on the recent integrated genomic characterization of endometrial cancer, its classification into four categories has been suggested: (i) cancers with mutations in DNA polymerase ε; (ii) hypermutated cancers with microsatellite instability; (iii) cancers with low frequency of DNA amplifications; and (iv) cancers with high frequency of DNA amplifications (Kandoth et al., 2013). The first three groups comprise endometrioid endometrial cancers, and the last group includes serous and endometriod types of endometrial cancers (Kandoth et al., 2013). The endometrioid types have usually been considered as type I endometrial cancers, while the poorly differentiated endometrioid endometrial cancer (grade 3) was recently classified as type II endometrial cancer (Murali et al., 2014).

Epidemiological studies have identified several risk factors for the development of endometrial cancer, which include obesity (Jenabi and Poorolajal, 2015), estrogen-only therapy, early menarche, late menopause, and nulliparity, among others. Recent studies have shown that both type I and type II endometrial cancers share several risk factors (Setiawan et al., 2013), and patients with these cancers show no difference in E2 and progesterone blood levels, which suggest similar pathogenesis (Wan et al., 2016). Obesity is an important risk factor for the development of endometrial cancer. It is associated with higher levels of circulating estrogens in postmenopausal women, as adipose tissue can serve as a source of estrogens that are formed from inactive precursors of adrenal or ovarian origin (Blouin et al., 2009). Additionally, the high-risk population includes patients treated with tamoxifen. This is the standard therapy for the majority of the 1.6 million breast cancer patients identified yearly worldwide (Ferlay et al., 2013), and also for patients with Lynch syndrome, with over one million cases in Europe alone (Hampel and de la Chapelle, 2011).

Most of the risk factors for the development of endometrial cancer can be explained by the unopposed estrogen hypothesis. According to this hypothesis, the exposure to endogenous or exogenous estrogens in the absence of progesterone or synthetic progestins increases the proliferation of endometrial cells and the concurrent DNA replication errors. This can result in somatic mutations and malignant transformations (Henderson and Feigelson, 2000; Akhmedkhanov et al., 2001). In endometrial cancer, estrogens drive proliferation *via* estrogen receptor α (ERα), which belongs to the superfamily of nuclear receptors and is encoded by *ESR1*. The presence of ERα is related to early-stage cancer, while a shift in the ratio between ERα and estrogen receptor β (ERβ) or loss of ERα are associated with shorter disease-free survival (Saegusa and Okayasu, 2000; Sakaguchi et al., 2002; Hu et al., 2005; Mylonas, 2010; Zannoni et al., 2013). Progesterone opposes the action of estrogens and stimulates differentiation, as supported by association of the genetic variations in genes that encode the PRA and PRB progesterone receptors with increased risk of endometrial cancer (Lee et al., 2010).

Epidemiological evidence suggests that also elevated blood levels of androgens, including testosterone, androstenedione, and dehydroepiandrosterone-sulfate (DHEA-S), are associated with greater risk of endometrial cancer (Lukanova et al., 2004). Significantly increased serum concentrations of DHEA, androstenedione, and testosterone were seen in patients with type I endometrial cancer when compared to healthy women (Audet-Walsh et al., 2011). Interestingly, an epidemiological study in premenopausal women revealed no associations between androgens and endometrial cancer (Clendenen et al., 2016). The importance of androgens as etiological factors has been supported also by the expression of the androgen receptor and the presence of androgen-metabolizing enzymes in well and moderately differentiated endometrial cancer (Gibson et al., 2014). However, in contrast to epidemiological studies, these *in-vitro* reports have suggested protective effects of androgens. Currently, the role of androgens in endometrial cancer is thus not well-defined, although higher blood concentrations of androgens seen in patients with type I endometrial cancer, together with the presence of the androgen receptor and androgen-metabolizing enzymes in tissue samples, have suggested that androgens do not serve only as precursors of estrogens, but probably have discrete roles in the pathophysiology of this gynecological cancer.

### Ovarian cancer and steroid hormones

Ovarian cancer is a heterogeneous disease that encompasses five major types of tumors that show different etiologies, risk factors, origins, molecular features, and clinical behaviors. These tumors are mainly derived from non-ovarian tissues that have colonized the ovaries. As much as 90% of all ovarian cancers are epithelial ovarian cancers. With a frequency of 70%, the most common ovarian cancer is high-grade serous carcinoma (which originates from serous tubal intraepithelial carcinomas in the Fallopian tubes). This is followed by endometrioid carcinoma and clear-cell carcinoma (both of which originate from endometrial cells), at 10% frequency each, and then low-grade serous carcinoma (which originates from benign lesion in the ovary) and mucinous carcinoma (which originates from gastrointestinal tissue), at 5% of all epithelial cancers (Binder et al., 2015; Prat, 2015; Ramalingam, 2016). High-grade serous ovarian carcinoma carries *TP53* mutations, while low-grade serous ovarian carcinoma has wild-type *TP53*, but mutations in *K-RAS, B-RAF*, and other oncogenes (Rojas et al., 2016). Most patients with ovarian cancer are diagnosed with advanced stage disease and consequently have poor prognosis. Only 46% of these patients survive for 5 years, and compared to other cancers, the overall survival has not increased significantly in the last 40 years (Bast, 2011).

Data from epidemiological studies suggest that ovarian cancer depend on estrogens, although the precise roles of estrogens have not yet been defined (Chura et al., 2009a). Epidemiological studies (Women's Health Initiative and Million Women Study) (Anderson et al., 2003; Beral et al., 2007, 2015) have indicated that both estrogen only and combined estrogen–progestin hormone replacement therapies increase the risk of serous and endometrioid ovarian cancer, but not of other types. Also, genetic susceptibility studies have supported estrogens in the etiology of ovarian cancer, as a single nucleotide polymorphism (SNP) in the *ESR2* gene, which codes for ERβ, and which is considered a tumor suppressor, is associated with significantly increased risk of ovarian cancer (Lurie et al., 2011). Moreover, in clinical samples of ovarian cancer, ERα is widely expressed, while the levels of ERβ expression are progressively lost during ovarian cancer progression toward metastatic tumors (Rutherford et al., 2000). Estrogens have also been reported to stimulate ovarian cancer proliferation and to increase metastatic potential (Bai et al., 2000; Park et al., 2008).

The observed beneficial effects of pregnancy and increased incidence of ovarian carcinoma among women with progesterone deficiency (Diep et al., 2015) imply that progesterone and progestins might prevent the development of ovarian cancer (Modan et al., 1998; Ho, 2003; Modugno et al., 2012; Jeon et al., 2016). Protective effects of progesterone have also been supported by the reduced PRA levels in ovarian carcinoma compared to benign ovarian tissue (Ho, 2003). On the other hand, a SNP in the *PGR* gene that influences the levels of the encoded PRA and PRB does not affect the risk of ovarian cancer (Pearce et al., 2008; Modugno et al., 2012). Some epidemiological studies have indicated that circulating androgens might also have roles in ovarian cancer, while other studies have not supported androgens as an etiological factor (for review, see Modugno et al., 2012).

The epidemiological data on the involvement of steroid hormones in the etiology of ovarian cancer are thus currently inconclusive, and in some cases contradictory. As ovarian cancer predominately affects women after menopause, it might be explained by a greater importance of steroid biosynthesis from adrenergic precursors and their actions in tumor tissue compared to the circulating hormone levels (Modugno et al., 2012). This is supported by the presence and activity of androgen and estrogen biosynthetic enzymes in epithelial ovarian cancer (Chura et al., 2009b).

## Intracrine actions of steroid hormones

In higher primates and humans, steroid hormones act in endocrine, paracrine, autocrine, and intracrine manners. In these species, steroid hormones are formed in the endocrine glands and also in peripheral tissues, from inactive precursors of adrenal origin or *de-novo* from cholesterol. The biosynthesis and actions of steroid hormones in humans thus differ importantly from the situation in animal models, where steroid hormones are formed exclusively in the endocrine glands and act in the target tissues (Luu-The and Labrie, 2010). In humans, steroid hormones thus act in the same (i.e., intracrine, autocrine) or the neighboring (i.e., paracrine) cell(s) where they are formed. Steroid hormones that are formed in a particular cell can activate the corresponding intracellular receptors of the nuclear receptor superfamily, which act as transcription factors. The activated receptor dimer then binds to the hormone-responsive elements of DNA, which is followed by binding of co-activators/co-repressors and other indispensable components of the transcription machinery. In this manner, steroid hormones regulate the expression of the target genes over a time period of hours or days (for review, see Hewitt et al., 2016). As genes that encode steroid hormone receptors have several transcripts and splice variants, this brings additional complexity to their mechanisms of action (for review, see Prossnitz and Barton, 2011; Thomas and Gustafsson, 2011; Hattori et al., 2016).

Steroid hormones can also activate membrane-bound receptors, in terms of the covalently modified, palmitoylated classical receptors of the nuclear receptor superfamily (Levin, 2011), or G-protein-coupled receptors (Prossnitz et al., 2008), or newly identified membrane receptors (Romero-Sánchez et al., 2008). In this manner, they activate intracellular signaling pathways, and can thus have rapid effects that occur in the time frame of minutes or hours.

It has been known for more than 30 years that steroid hormones can be formed from inactive steroid precursors in target peripheral tissues. The terms intracrine action and intracrinology were coined by Labrie to describe the mechanism of action where steroids are formed in the same cell in which they exert their action (Labrie, 1991). Active androgens and estrogens can be formed from inactive or less active precursor steroid hormones, mainly DHEA, DHEA-S, androstenedione, and estrone-sulfate (E1-S). The local formation of steroid hormones thus has major roles in both normal tissues, such as breast, muscle, skin, and bone (Suzuki et al., 2005), and also in hormone-dependent tumor tissues, which comprise up to 20% of all cancers (Ferlay et al., 2013).

### DHEA-S as a precursor for androgen and estrogen formation

Humans and other primates are unique in that their adrenal glands (*zona reticularis*) produce large quantities of the inactive steroid precursors DHEA, DHEA-S, and androstenedione, which can be metabolized in peripheral tissues into active androgens and estrogens. These reactions depend on the presence of androgen and estrogen forming and inactivating enzymes (Labrie et al., 1998, 2015). In this manner peripheral target tissues can control and adjust the formation and inactivation of steroid hormones according to local requirements.

Labrie et al. (1998) estimated that 30–50% of androgens in men in their 50 and 60 s are formed in peripheral tissues from inactive precursors from the adrenal gland, mainly as DHEA and DHEA-S. In women after menopause, intracrine formation is even more important, with up to 100% of estrogens formed from the adrenal precursors DHEA and DHEA-S, and *de-novo* synthesis of the androgen androstenedione from cholesterol in the ovaries (Labrie et al., 1998; Fogle et al., 2007).

Serum concentrations of DHEA change through life, with production of DHEA and DHEA-S increasing during adrenarche, after the age of 6–8 years. These increased circulating levels of DHEA and DHEA-S are then maintained for two decades, before they start to decline after the third decade of life (Labrie et al., 1997). The levels of DHEA thus regress with advanced age, where the rate of this regression differs among races; e.g., lower decreases in circulating DHEA-S levels have been reported in Japanese women compared to Caucasians (Crawford et al., 2009). In spite of this decline, the plasma concentrations of DHEA and DHEA-S in adult men and women are still 200–3,000-fold higher than those of testosterone, 2,000–20,000-fold higher than those of 5α-dihydrotestosterone (5αDHT), and 30,000–800,0000-fold higher than those of estradiol (E2) (Labrie et al., 1998; Audet-Walsh et al., 2011; Giton et al., 2011) (Table 1), with large inter-individual variability seen. Interestingly, increased concentrations of DHEA-S have been reported for menopausal transition, which might be related to the rise of luteinizing hormone (Yasui et al., 2012).

**Table 1:** Serum steroid hormone levels in healthy premenopausal and postmenopausal women.

**Steroid hormone Mw (g/mol)**	**Pre-menopausal women**			**Post-menopausal women**		
	**Age (years)/BMI (kg/m^2^)**	**Concentration (number of women)**	**Reference**	**Age (years)/BMI (kg/m^2^)**	**Concentration (number of women)**	**Reference**
DHEA-S	30–35/–	1.27 ± 0.62 μg/ml	Labrie et al., 2006	55–65/–	0.59 ± 0.36 μg/ml	Labrie et al., 2006
(368.6)		**3.44 ± 1.68 μM**			**1.60 ± 0.98 μM**	
		(47)			(377)	
		(mean ±*SD*)			(mean ±*SD*)	
	30 (19–40)/	1.94 μg/ml	Keefe et al., 2014	58.3 ± 5.6/27.0 ± 5.4	0.60 (0.23–1.29) μg/ml	Audet-Walsh et al., 2011
	27.8 (21.1–33.3)	(0.36–3.78)		(mean ±*SD*)	**1.63 (0.62–3.50) μM**	
	(2.5, 97.5th percentile)	**5.26 μM**			(110)	
		(0.97–10.26)			(10–90th percentile)	
		(42, folicular phase)				
		(2.5, 97.5th percentile)				
DHEA	30–35/–	4.47 ± 2.19 ng/ml	Labrie et al., 2006	55–65/–	1.95 ± 1.18 ng/ml	Labrie et al., 2006
(288.4)		**15.50 ± 7.59 nM**			**6.76 ± 4.09 nM**	
		(47)			(377)	
		(mean ±*SD*)			(mean ±*SD*)	
	30 (19–40)/27.8 (21.1–33.3)	3.89 ng/ml	Keefe et al., 2014	58.3 ± 5.6/27.0 ± 5.4	1.91 (0.84–4.34) ng/ml	Audet-Walsh et al., 2011
	(2.5, 97.5th percentile)	(0.67–10.94)		(mean ±*SD*)	**6.62 (2.91–15.05) nM**	
		**13.49 (2.32–37.93) nM**			(110)	
		(42, folicular phase)			(10–90th percentile)	
		(2.5, 97.5th percentile)				
A-dione	30–35/–	0.96 ± 0.35 ng/ml	Labrie et al., 2006	55–65/–	0.40 ± 0.18 ng/ml	Labrie et al., 2006
(286.4)		**3.35 ± 1.22 nM**			**1.39 ± 0.63 nM**	
		(47)			(377)	
		(mean ±*SD*)			(mean ±*SD*)	
	30 (19–40)/27.8 (21.1–33.3)	1.06 ng/ml	Keefe et al., 2014	58.3 ± 5.6/27.0 ± 5.4	0.44 (0.24–0.80) ng/ml	Audet-Walsh et al., 2011
	(2.5, 97.5th percentile)	(0.69–2.23)		(mean ±*SD*)	**1.54 (0.84–2.79) nM**	
		**3.70 (2.41–7.79) nM**			(110)	
		(42, folicular phase)			(10–90th percentile)	
		(2.5, 97.5th percentile)				
Testosterone	30–35/–	0.18 ± 0.07 ng/ml	Labrie et al., 2006	55–65/–	0.14 ± 0.07 ng/ml	Labrie et al., 2006
(288.4)		**0.62 ± 0.24 nM**			**0.49 ± 0.24 nM**	
		(47)			(377)	
		(mean ±*SD*)			(mean ±*SD*)	
	30 (19–40)/27.8 (21.1–33.3)	0.242 ng/ml	Keefe et al., 2014	58.3 ± 5.6/27.0 ± 5.4	0.14 (0.06–0.24) ng/ml	Audet-Walsh et al., 2011
	(2.5, 97.5th percentile)	(0.10–0.588)		(mean ±*SD*)	**0.49 (0.21–0.83) nM**	
		**0.76 (0.35–2.04) nM**			(110)	
		(42, folicular phase)			(10–90th percentile)	
		(2.5, 97.5th percentile)				
DHT	30–35/–	70 ± 30 pg/ml	Labrie et al., 2006	55–65/–	40 ± 30 pg/ml	Labrie et al., 2006
(290.4)		**241.05 ± 103.31 pM**			**137.74 ± 103.31 pM**	
		(47)			(377)	
		(mean ±*SD*)			(mean ±*SD*)	
		82.12 ± 25.10 pg/ml	Caron et al., 2009	58.3 ± 5.6/27.0 ± 5.4	30.00 (10.00–70.00) pg/ml	Audet-Walsh et al., 2011
		**282.78 ± 86.43 pM**		(mean ±*SD*)	**103.31 (34.44–241.05) pM**	
		(10)			(110)	
		(mean ±*SD*)			(10–90th percentile)	
Estrone	30–35/–	53.96 ± 23.28 pg/ml	Labrie et al., 2009	58.3 ± 5.6/27.0 ± 5.4	18.36 (10.01–35.45) pg/ml	Audet-Walsh et al., 2011
(270.4)		**199.56 ± 86.09 pM**			**67.90 (37.02–131.10) pM**	
		(47)		(mean ±*SD*)	(110)	
		(mean ±*SD*)			(10–90th percentile)	
	32.1 ± 7.9/–	38.50 ± 11.86 pg/ml	Caron et al., 2009	55–74/–	14.59 (13.67–16.07) pg/ml	Fuhrman et al., 2012
	(mean ±*SD*)	**142.38 ± 43.86 pM**			**53.95 (50.54–59.43) pM**	
		(19, follicular)			(423)	
		75.84 ± 31.62 pg/ml			(median, 10–90th percentile)	
		**280.47 ± 116.94 pM**				
		(19, luteal)				
		(mean ±*SD*)				
Estradiol	30–35/–	82.05 ± 42.19 pg/ml	Labrie et al., 2009	58.3 ± 5.6/27.0 ± 5.4	3.35 (1.00–9.67) pg/ml	Audet-Walsh et al., 2011
(272.4)		**301.21 ± 154.88 pM**		(mean ±*SD*)	**12.30 (3.67–35.50) pM**	
		(47)			(110)	
		(mean ±*SD*)			(10–90th percentile)	
	32.1 ± 7.9/–	38.40 ± 20.40 pg/ml	Caron et al., 2009	55–74/–	4.21 (3.96–4.57) pg/ml	Fuhrman et al., 2012
	(mean ±*SD*)	**140.97 ± 74.89 pM**			**15.45 (14.54–16.77) pM**	
		(19, follicular)			(423)	
		103.66 ± 73.27 pg/ml			(median, 10–90th percentile)	
		**380.54 ± 268.98 pM**				
		(19, luteal)				
		(mean ±*SD*)				
Estrone-S	32.1 ± 7.9/–	0.64 ± 0.37 ng/ml	Caron et al., 2009	55–65/–	0.22 ± 0.01 ng/ml	Labrie et al., 2009
(350.4)	(mean ±*SD*)	**1.83 ± 1.06 nM**			**0.63 ± 0.03 nM**	
		(19, follicular)			(377)	
		1.92 ± 1.09 ng/ml				
		**5.48 ± 3.11 nM**				
		(19, luteal)				
		(mean ±*SD*)				
				58.3 ± 5.6/27.0 ± 5.4	0.17 (0.04–0.52) ng/ml	Audet-Walsh et al., 2011
				(mean ±*SD*)	**0.49 (0.11–1.48) nM**	
					(110)	

*All steroid hormone concentrations included in this table were measured by GC-MS or LC-MS/MS. Molar concentrations are in bold*.

**Figure 1:**
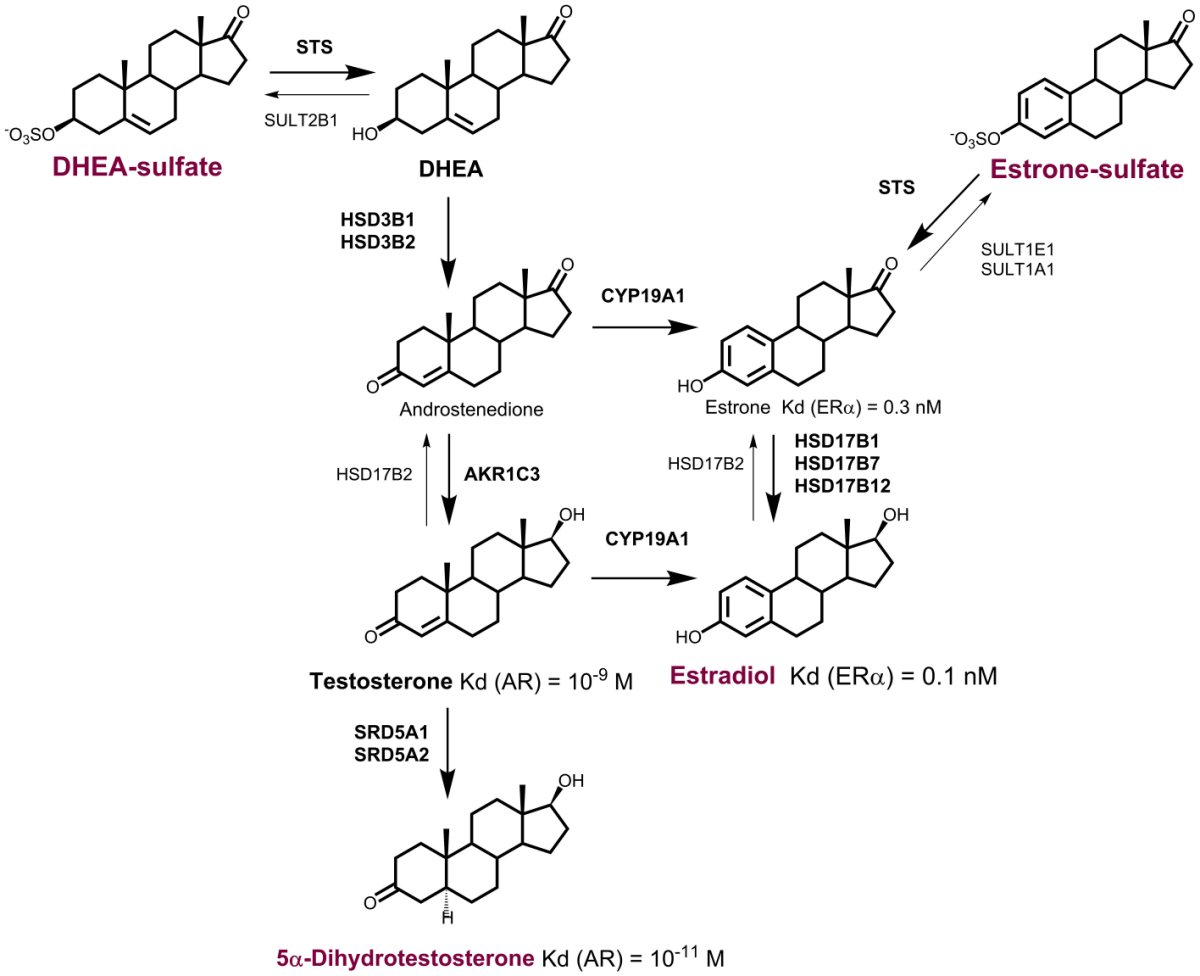
Formation of active steroid hormones from DHEA-S and E1-S. In peripheral tissues, dehydroepiandrosterone sulfate (DHEA-S), and estrone-sulfate (E1-S) serve as precursors for formation of active steroid hormones. DHEA-S can be activated to estradiol (E2) via androstenedione by the actions of sulfatase (STS), 3β-hydroxysteroid dehydrogenases type 1 and 2 (HSD3B1, HSD3B2), aromatase (CYP19A1), and the reductive 17β-hydroxysteroid dehydrogenases (HSD17B1, HSD17B7, 17HSD12) or aldo-keto reductase (AKR1C3). E2 can also be formed from estrone sulfate (E1-S) by the action of sulfatase (STS) and reductive HSD17B. DHEA-S can be activated to active androgens. The most potent androgen 5α-dihydrotestosterone can be formed from DHEA-S by the actions of STS and HSD3B1, or HSD3B2 and AKR1C3, and 5α-reductases types 1 and 2 (SRD5A1, SRD5A2).

In postmenopausal women, the daily production of DHEA is 6–8 mg, with about 50% of this originating from the adrenal gland, 20% from the ovarian theca cells, and the remaining 30% from the circulating DHEA-S, through the action of sulfatase (Yasui et al., 2012). In these women, plasma concentrations of DHEA and DHEA-S are relatively high; at approximately 6.6 nM and 1.6 μM, respectively (Table 1). In contrast, plasma concentrations of E1 and E2 are much lower, at 60 and 12–15 pM, respectively. DHEA and DHEA-S can serve as sources for the local formation of androgens and estrogens. In peripheral tissues, E2 can be synthesized from adrenal DHEA and DHEA-S, and also from adrenal or ovarian androstenedione (Figure 1). Androstenedione formed from DHEA or DHEA-S can be further activated to testosterone, by the action of 17-ketosteroid reductase type 5, which is better known as aldo-keto reductase 1C3 (AKR1C3). Testosterone can also be further activated to the most potent androgen 5α-DHT, by 5α-reductases type 1 and 2 (Figure 1). In patients with endometrial cancer, plasma DHEA, and androstenedione concentrations are increased (Audet-Walsh et al., 2011) and are related to increased risk of endometrial cancer (Potischman et al., 1996; Lukanova et al., 2004). Also, a strong association between DHEA-S and breast cancer risk has been reported (Key et al., 2002), while no association with DHEA or androstenedione was seen for ovarian cancer (Modugno et al., 2012).

### Estrone sulfate as a precursor for estrogen formation

Estrone-sulfate (E1-S) represents the most abundant estrogen in the peripheral blood and an important steroid precursor for estrogen formation after activation by sulfatase (STS) and 17-ketosteroid reductases (HSD17B1, HSD17B7, or HSD17B12) (Figure 1), with relatively high concentrations in postmenopausal women (0.5–0.6 nM). E1-S levels are associated with high body mass index (Jasonni et al., 1984), which implies that E1-S originates mainly from adipose tissue. Indeed, high concentrations of E1-S have been reported for visceral adipose tissue as compared to blood, where local concentrations are up to 60-fold higher compared to serum (Labrie, 2003).

About 3-fold higher E1-S plasma concentrations were reported for postmenopausal patients with endometrial cancer type I, compared to control healthy women, while no significant difference was seen between endometrial cancer type II and healthy women (Audet-Walsh et al., 2011). This suggests that in type I endometrial cancer, E1-S might represent a source for local E2 formation, or might reflect increased metabolism of estrogens in cancer tissue. The levels of E1-S are significantly decreased in patients with endometrial cancer with less-differentiated tumors and in patients with myometrial invasion and lympho-vascular space invasion. On the other hand, the circulating levels of E1-S are 2-fold higher in recurrent cases compared to non-recurrent cases (Audet-Walsh et al., 2011). Also, in ovarian cancer, E1-S may serve as a precursor for biosynthesis of E2. Chura et al. (2009a) reported that the great majority of epithelial ovarian cancer samples show E1 sulfatase activity and 17-ketosteroid reductase activity, which promote the activation of E1-S to the most potent estrogen E2. Additionally, ovarian cancer cell lines have capacity for metabolism of E1-S to E1 and E2 (Ren et al., 2015).

DHEA-S and E1-S can serve as precursors for active steroid hormones. Although the levels of DHEA-S and E1-S decrease after menopause, serum concentrations of these precursors in postmenopausal women are sufficient for the local formation and actions of steroid hormones. To reach the sites of intracrine or paracrine action, DHEA-S and E1-S have to cross several biological membranes. First, they have to be extruded from their site of synthesis, and when they reach the peripheral tissue, they have to be taken up by individual cells.

### Transporters for DHEA-S and E1-S

The key determinants of the efflux and uptake of DHEA-S and E1-S are the membrane proteins known as ATP-binding-cassette (ABC)-transporters (Ween et al., 2015), organic anion-transporting polypeptides (OATPs) (Obaidat et al., 2012; Roth et al., 2012), organic anion transporters (OAT) (Burckhardt and Burckhardt, 2011), and transporters encoded by the members of solute carrier family 51 (*SLC51*) (Ballatori et al., 2013) (Table 2). In peripheral tissues, these uptake transporters have crucial roles in providing the steroid precursors for local androgen and estrogen formation.

**Table 2:** Characteristics of the most relevant E1-S and DHEA-S transporters.

**Family**	**Protein**	**Gene**	**K_m_ for E1-S (μM)**	**Reference**	**K_m_ for DHEAS (μM)**	**Reference**
OATPs	1A2	SLCO1A2	16–59	Lee W. et al., 2005	7	Kullak-Ublick et al., 1998
	1B1	SLCO1B1	0.3–45	Tamai et al., 2001; Roth et al., 2012	22	Kullak-Ublick et al., 2001; Roth et al., 2012
	1B3	SLCO1B3	58	Gui et al., 2008	>30	Cui et al., 2001
	2B1	SLCO2B1	21	Tamai et al., 2001; Pizzagalli et al., 2003	9	Pizzagalli et al., 2003
	4A1	SLCO4A1	n.d.	Tamai et al., 2000	–	–
	4C1	SLCO4C1	26.6	Yamaguchi et al., 2010	–	–
OATs	OAT2	SLC22A7	n.d.	Kobayashi et al., 2005	–	–
	OAT3	SLC22A8	2.2 and 21.2	Burckhardt and Burckhardt, 2011	13	Nozaki et al., 2007
	OAT4	SLC22A11	1.01 and 21.7	Burckhardt and Burckhardt, 2011	0.63 and 29.2	Burckhardt and Burckhardt, 2011
	OAT7	SLC22A9	8.7	Burckhardt and Burckhardt, 2011	2.2	Burckhardt and Burckhardt, 2011
ABC	ABCC1/MRP1		0.9	Conrad et al., 2002	5	Zelcer et al., 2003
	ABCC4/MRP4		–		2	Zelcer et al., 2003
	ABCC11/MRP8		–	Arlanov et al., 2016	21	Bortfeld et al., 2006
	ABCG2/BCRP		6.8	Imai et al., 2003	–	Lee Y. J. et al., 2005
SLC51	OSTα/β	SLC51A/B	n.d.	Ballatori et al., 2005	n.d.	Ballatori et al., 2005

n.d.: not measured

### Organic anion-transporting polypeptides

The OATPs are encoded by genes of the solute carrier for organic anions *(SLCO)* family, where this OATP/*SLCO* family comprises 11 members in humans. OATPs have been detected in various cells and tissues of the human body (Roth et al., 2012). Some OATPs, like OATP1A2, OATP2B1, and OATP4A1, are ubiquitous, while OATP1B1 and OATP1B3 are restricted to hepatocytes (Hagenbuch and Stieger, 2013). However, under pathological conditions, such as in cancers, the OATP expression pattern is changed (Obaidat et al., 2012). OATPs are anion exchangers that mediate the cellular uptake of large (>300 Da) organic, mostly negatively charged, molecules in a Na^+^- and ATP-independent manner (Roth et al., 2012). Their physiological substrates are bilirubin, bile acids, prostaglandins, thyroid hormones, and conjugated steroid hormones, such as DHEA-S and E1-S. OATP1A2, OATP1B1, OATP1B3, and OATP2B1 mediate the uptake of DHEA-S, while OATP1A2, OATP1B1, OATP1B3, OATP2B1, OATP4A1, and OATP4C1 catalyze the uptake of E1-S (Table 2). At least four members of the family (i.e., OATP1A2, OATP1B1, OATP1B3, OATP2B1) transport various clinically applied drugs, in addition to endobiotics. Therefore, these proteins are also key determinants of drug absorption, distribution, and excretion (Kovacsics et al., 2016).

### Organic anion transporters

The OATs are anion exchangers that are encoded by members of the *SLC22* gene family. Most OATs are polyspecific, as they can transport structurally diverse, relatively hydrophilic, low molecular mass (<500 Da) compounds. Several members of the *SLC22* gene family, including OAT1 and OAT3, are well-established determinants of renal clearance, intestinal absorption, and hepatic elimination of drugs (Burckhardt and Burckhardt, 2011; Koepsell, 2013). On the other hand, OATs are also important in neurotransmitter homeostasis in the brain, and at least four members of this family (i.e., OAT2, OAT3, OAT4, OAT7) transport E1-S, while OAT3, OAT4, and OAT7 transport DHEA-S (Koepsell, 2013) (Table 2). OAT2 is expressed in the liver and kidney (Koepsell, 2013), and in adipose tissue (Human Protein Atlas). OAT3 is expressed in the kidney, and also in the blood-brain barrier (Burckhardt and Burckhardt, 2011), while OAT4 localizes to the kidney, and is also present in the placenta, where it mediates the uptake of DHEA-S that is crucial for placental estrogen synthesis (Ugele et al., 2003).

### Transporters encoded by solute carrier *(SLC) 51* gene family (OSTα, OSTβ)

The human *SLC51* gene family has only two members, *SLC51A* and *SLC51B*, which are also known as organic solute transporter (OST)α and OSTβ (Ballatori et al., 2013). OSTα and OSTβ are obligate heterodimers that transport bile acids and conjugated steroids; i.e., E1-S and DHEA-S. OSTα and OSTβ act down the concentration gradient of their substrates, and therefore they may be involved in both efflux and uptake processes (Ballatori et al., 2013, 2008). In humans, the *SLC51A* and *SLC51B* mRNAs have been reported for many tissues, including intestine, kidney, liver, testes, ovary, uterus, prostate, adrenal, and mammary gland (Seward et al., 2003).

### ATP-binding-cassette transporters

There are 48 distinct ABC-transporters in humans, which have been grouped into seven subfamilies, from ABC-A to ABC-G (Ween et al., 2015). The ABC-transporters mediate the active transport of various compounds across extracellular and intracellular membranes, with the energy derived from hydrolysis of ATP. Many ABC-transporters work as efflux pumps, to extrude their substrates from cells. ABC-transporters recognize a large variety of endogenous substances, and also chemically distinct molecules, including clinically applied drugs (Sarkadi et al., 2006). Overexpression of polyspecific ABC-transporters in tumors results in increased extrusion of drugs and can lead to resistance against multiple anti-cancer agents, termed as multi-drug resistance (MDR) (Schinkel and Jonker, 2003). The three major ABC-transporters implicated in MDR are ABCB1 (Pgp, P-glycoprotein) (Sharom, 2014), ABCC1, which is better known as MDR-associated protein 1 (MRP1) (Cole, 2014), and ABCG2, which is also known as breast cancer resistance protein (BCRP) or mitoxantrone resistance protein (MXR) (Ishikawa and Nakagawa, 2009). The multispecific MDR transporters are proven determinants of drug absorption, distribution, and excretion (Szakacs et al., 2008).

Members of ABC-transporter family C (i.e., ABCC1, ABCC4, ABCC11, ABCG2) transport large, negatively charged molecules. ABCC1 (MRP1) is ubiquitous in the body, and its main physiological substrates are leukotriene C4, and various glutathione-conjugated, glucuronidated, or sulfated compounds, including E1-S (Bodo et al., 2003; Cole, 2014). ABCC4 (MRP4) is found throughout the human body and transports molecules that are involved in cellular signaling, such as cyclic nucleotides, eicosanoids, and conjugated steroids (Slot et al., 2011). ABCC11 (MRP8) is found in brain, breasts, lungs, liver, kidney, placenta, prostate, testes, and apocrine glands, as well as in cancerous tissues (Kruh et al., 2007). Its substrates are lipophilic anions, as well as cyclic nucleotides and anticancer drugs (Bortfeld et al., 2006; Matsumoto et al., 2014). ABCC1, ABCC11, and ABCG2 transport E1-S, while ABCC1, ABCC4, ABCC11, and ABCG2 mediate excretion of DHEA-S (Table 2).

Concerted (and possibly co-regulated) actions of OATPs, OATs, transporters encoded by the *SLC51* gene family, ABC-efflux pumps, and biotransformation enzymes are needed for the handling of potentially toxic endogenous (e.g., bile acids) and exogenous (e.g., drugs) compounds (Sarkadi et al., 2006). Similarly, united actions of these transporters and the intracellular enzymes are required for maintenance of normal steroid hormone homeostasis.

## Disturbed transport and estrogen actions in gynecological cancers

### Changes in uptake and excretion of steroid conjugates

Altered expression of OATPs (usually as up-regulation) has been documented in different types of cancers. As these transporters serve as mediators of the uptake of nutrients for tumor growth and survival, and as they also bring anticancer agents into cancer cells, they may have significant impact on cancer therapies (Li and Shu, 2014). High levels of the steroid transporting OATPs (i.e., OATP1A2, OATP2B1, OATP1B3, OATP4A1, OATP4C1) have been suggested for breast cancer cells, as compared to normal tissue (Pizzagalli et al., 2003; Al Sarakbi et al., 2006; Meyer zu Schwabedissen et al., 2008; Wlcek et al., 2008). OATP1B3 has also been observed in endometrial carcinoma, where high levels significantly correlated with type I tumors and longer disease-free survival (Ogane et al., 2013) (Table 3). High levels of OATP1B3 and lower levels of OATP1B1 have also been reported in ovarian cancer samples (Arakawa et al., 2012; Furihata et al., 2015; Thakkar et al., 2015) and cancer cell lines (Cho et al., 2009; Chay et al., 2010; Svoboda et al., 2011; Lancaster et al., 2013) (Table 3). In addition to OATP1B3 and OATP1B1, also OATP2B1, OATP3A1, and OATP4A1 have been detected at the mRNA level in ovarian cancer samples (Tamai et al., 2000).

**Table 3:** DHEA-S and E1-S transporters and estrogen biosynthetic enzymes in endometrial cancer.

**Gene**	**Level**	**Cell line**		**Tumor tissue**	
		**Regulation**	**Reference**	**Regulation**	**Reference**
*OATP1B3*	Protein			Cancer/adjacent tissue ↑	Ogane et al., 2013
*ABCC4*	mRNA	β HEC-1A after down-regulation of KLF9	Simmen et al., 2008		
*ABCG2*	mRNA	CD+133 Ishikawa cells, Expressed	Nakamura et al., 2014		
	Protein	CD+133 Ishikawa cells, Expressed	Nakamura et al., 2014		
*HSD3B1*	mRNA	HHUA, Expressed	Sugawara et al., 2004	Cancer/adjacent tissue ≈	Sinreih et al., 2013
		HIEEC, Ishikawa, HEC-1A Expressed ↑ HEC-1A/HIEEC	Hevir-Kene and Rižner, 2015		
*HSD3B2*	mRNA	HOUA, Expressed	Sugawara et al., 2004	Cancer/adjacent tissue ≈	Sinreih et al., 2013
		HIEEC, Ishikawa, HEC-1A Expressed ↑ HEC-1A/HIEEC	Hevir-Kene and Rižner, 2015		
*CYP19A1*	mRNA	HHUA, HOUA, No expression	Sugawara et al., 2004	Cancer/adjacent tissue, Low expression, ≈	Pathirage et al., 2006; Smuc and Rizner, 2009; Lépine et al., 2010; Cornel et al., 2012
		HIEEC, Ishikawa, HEC-1A Low expression ↑ HEC-1A/Ishikawa	Hevir-Kene and Rižner, 2015		
	Protein			Cancer/pre-/post-menopausal endometrium, No staining	Human Protein Atlas
	Activity	HEC-1, HEC-1B, RL-95, Ishikawa; No activity	Fournier and Poirier, 2009		
		HIEEC, Ishikawa, HEC-1A, No activity	Hevir-Kene and Rižner, 2015		
*HSD17B1*	mRNA	HIEEC, Ishikawa, HEC-1A Expressed, ↑ HEC-1A/HIEEC	Hevir-Kene and Rižner, 2015	Cancer/adjacent tissue, Low levels	Smuc et al., 2006; Lépine et al., 2010; Cornel et al., 2012
				Decreased	Smuc and Rizner, 2009; Lépine et al., 2010
				Increased in G1, ERα	Cornel et al., 2012
	Protein			Cancer/pre-/postmenopausal endometrium: 9% Cancer, weak/negative/negative	Human Protein Atlas
*HSD17B7*	mRNA	HIEEC, Ishikawa, HEC-1A Expressed, ↑ HEC-1A/HIEEC	Hevir-Kene and Rižner, 2015	Cancer/adjacent tissue Decreased	Smuc and Rizner, 2009; Lépine et al., 2010
				Unchanged	Lépine et al., 2010; Cornel et al., 2012
	Protein			Cancer/pre-/postmenopausal endometrium: 45% Cancer, weak/weak/weak	Human Protein Atlas
*HSD17B12*	mRNA	HIEEC, Ishikawa, HEC-1A Expressed, ↑ HEC-1A/HIEEC	Hevir-Kene and Rižner, 2015	Cancer/adjacent tissue, Unchanged	Smuc and Rizner, 2009
				No significant difference	Cornel et al., 2012
				EC type 2; Increased	Lépine et al., 2010
	Protein			Cancer/pre-/postmenopausal endometrium: 45% Cancer, weak/weak/weak	Human Protein Atlas
*HSD17B2*	mRNA	HIEEC, Ishikawa, HEC-1A Expressed ≈	Hevir-Kene and Rižner, 2015	Cancer/adjacent tissue, EC type 2; Increased	Lépine et al., 2010
				EC type 1; ≈ Increased in G2	Cornel et al., 2012
				EC type 1 postmenopausal; Increased	Lépine et al., 2010; Sinreih et al., 2013
	Protein			Cancer/pre-/postmenopausal endometrium; Moderate/moderate/moderate	Human Protein Atlas
*AKR1C3*	mRNA	HIEEC, Ishikawa, HEC-1A Expressed, ↑ Ishikawa, HEC-1A/HIEEC	Hevir-Kene and Rižner, 2015	Cancer/adjacent tissue; Increased in individual patients	Rizner et al., 2006
				No sign. difference	Smuc and Rizner, 2009
				Cancer/adjacent tissue; Increased trend in G2/G3 EC	Cornel et al., 2012
	Protein			Cancer/pre-/post-menopausal endometrium: 45% Cancer moderate/strong/strong	Human Protein Atlas
*STS*	mRNA	HIEEC, Ishikawa, HEC-1A Expressed	Hevir-Kene and Rižner, 2015	Cancer/adjacent tissue; ≈	Smuc and Rizner, 2009
				Increased	Lépine et al., 2010
	Protein			Cancer/pre-/postmenopausal endometrium: 82% Cancer weak-moderate/weak/weak	Human Protein Atlas
	Activity E1-S	HEC-1A, HEC-1B, RL-95, Ishikawa; Low activity in whole cells, higher activity in homogenates	Fournier and Poirier, 2009		
		AC-258	Milewich and Porter, 1987		
*SULT1E1*	mRNA	Control HIEEC, Ishikawa, HEC-1A Expressed ≈	Hevir-Kene and Rižner, 2015	Cancer/adjacent tissue type 1 and 2; Borderline increase	Lépine et al., 2010
				Cancer/adjacent tissue type 1 ≈	Hevir et al., 2011
	Protein			Cancer/pre-/postmenopausal endometrium: Not detected, Negative/moderate/negative, Not detected	Human Protein Atlas
*SULT2B1*	mRNA	HIEEC, Ishikawa, HEC-1A Expressed, ↑ Ishikawa and HEC-1A/HIEEC	Hevir-Kene and Rižner 2015	Cancer/adjacent tissue; Increased	Hevir et al., 2011
	Protein			Cancer/pre-/postmenopausal endometrium: Moderate/moderate/moderate	Human Protein Atlas

*Endometrial cancer cell lines: HHUA, Ishikawa, well differentiated cell lines; RL-95, moderately differentiated cell line; HOUHA, HEC-1A, HEC-1B poorly differentiated cell line; HIEEC: control cell line of normal endometrium (pre-menopausal)*.

Among the transporters encoded by the *SLC22* genes, OAT2 has been detected in colorectal cancer (Tashiro et al., 2014), while to date there are no studies on the expression of the *SLC22* genes that encode the OAT2, OAT3, OAT4, and OAT7 proteins, nor on the transporters from the OST/SLC51 family in endometrial and ovarian cancers.

ABC-transporters are commonly up-regulated in chemoresistant cancers, and increased expression and activity of these drug efflux pumps results in reduced cellular accumulation of drugs (Ween et al., 2015). In endometrial cancer, chemoresistance is the major problem in advanced and recurrent cases (Chaudhry and Asselin, 2009). However, the data on expression of *ABC-transporter*s in endometrial cancer are very limited. Among the steroid-transporting ABC-transporters, ABCG2 has been observed at the mRNA and protein levels in CD133^+^ Ishikawa endometrial cancer cells, which have characteristics of cancer stem cells (Nakamura et al., 2010, 2014), (Ween et al., 2015). Expression of *ABCC4* was detected in the HEC-1A endometrial cancer cell line, where the mRNA levels were suppressed by down-regulation of transcription factor *KLF9*. Decreased expression of *KLF9* has previously been observed in endometrial cancers of stages II to IV (Simmen et al., 2008).

In ovarian cancer, chemoresistance is typically observed after initial sensitivity to platin-based and taxol-based therapies. The great majority of patients with advanced ovarian cancer develop MDR due to the overexpression of the ABC-transporters (Auner et al., 2010). In ovarian cancer, expression of *ABCC1* has been observed at the mRNA and protein levels in serous, mucinous, clear-cell, endometrioid, and undifferentiated ovarian cancer tissues (for recent review, see Ween et al., 2015). Across multiple studies, positive immunohistochemical staining for ABCC1 (MRP1) has been seen in 22–68% of paraffin sections from ovarian cancers (for review. see Ween et al., 2015), with association with higher tumor grade reported (Bagnoli et al., 2013). Also *ABCC4* is expressed in ovarian cancers. In a study that included 127 patients with ovarian cancer, ABCC4 (MRP4) was associated with shorter progression-free survival (Bagnoli et al., 2013; Ween et al., 2015). Finally, ABCC11 and ABCG2 have also been detected in ovarian cancers; the ABCG2 as a marker of side-populations of ovarian cancer cells that show stem-cell characteristics (Ween et al., 2015). In the *ABCG2* gene, several SNPs with functional consequences have been described, although controversial data have been reported considering their association with the outcome of ovarian cancers (for review, see Ween et al., 2015). *ABCG2* was induced in patients after their chemotherapy, and also by estrogens in the PA-1 ovarian cancer cell line after stable transfection with the gene encoding ERα (for review, see Ween et al., 2015). Recent systematic genomic analysis and biological properties of ovarian cancer cell lines showed that many commonly used “ovarian cancer” cell lines are unlikely to originate from high-grade serous ovarian cancer (Domcke et al., 2013; Haley et al., 2016). Therefore, novel cancer cells lines recently established will provide better models (Kreuzinger et al., 2015).

Analysis of The Cancer Genome Atlas data for the *SLCO* and *SLC22* genes in endometrial and ovarian cancers (cbioportal; http://www.cbioportal.org) (Cerami et al., 2012; Gao et al., 2013) has provided further information. The *SLCO1A2, SLCO1B1, SLCO1B3, SLCO1C1, SLCO2B1*, and *SLCO4A1* genes were amplified in <1% of patients, but showed mutations (as mainly missense mutations), in 3.1–5.6% of 240 patients with endometrial cancer. A similar trend was seen for the *SLC22* genes. Mutations were detected in all four *SLC22A* genes (i.e., *SLC22A7, SLC22A8, SLC22A11, SLC22A9*), in 2.6–5.1% of all patients. Among the *ABC* genes, there were mutations in all four genes involved in the efflux of steroid conjugates (i.e., *ABCC1, ABCC4, ABCC11, ABCG2*), in 5.7–8.3% of patients with endometrial cancer. In contrast, the *SLCO1A2, SLCO1B1, SLCO1B3, SLCO1C1, SLCO2B1*, and *SLCO4A1* genes were amplified in 4.7–7.4% of a total of 316 patients with serous ovarian cancer, with concurrent amplification of *SLCO1A2*/*1B1/1B3/1C1* and *SLCO1B1/1B3/1C1*, and also *SLCO1B3/1C1*. Among the *SLC22* and *ABC* genes, *SLC22A7* and *ABCC1* were amplified in 3.5 and 2.5% of patients, respectively, with serous ovarian cancers.

**Figure 2:**
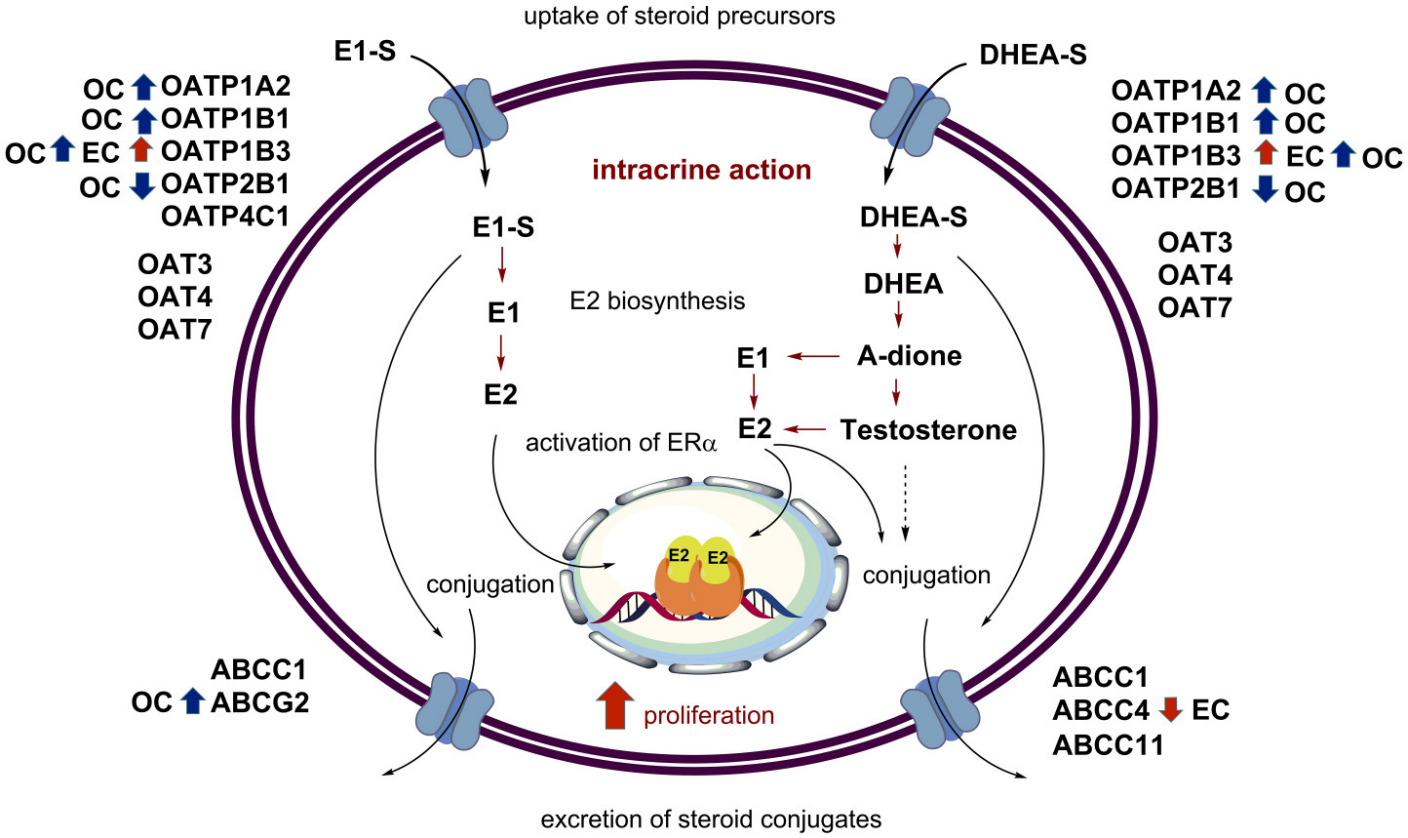
Uptake, intracrine action, and excretion of steroid hormones. The major players in intracrine estrogen action are depicted, with the additional data on the levels of OATP, OAT, and ABC-transporters in endometrial (red arrows) and ovarian cancers (blue arrows).

Although, the current knowledge on transporters in endometrial cancer are scarce, the currently available data suggest that E1-S and DHEA-S might enter cancerous cells via OATP1B3, which is present mainly in type I tumors (Ogane et al., 2013) (Table 3, Figure 2). This uptake might be affected by the missense mutations seen in 5.1% of patients (cbioportal; http://www.cbioportal.org). On the other hand, excretion of steroid conjugates can be catalyzed by ABCC4, a high affinity DHEA-S transporter that shows down-regulated expression in advanced stages of cancer (III-IV) (Simmen et al., 2008) (Table 4, Figure 2). Similarly, increased levels of ABCG2 were observed in CD133+ Ishikawa cells (Nakamura et al., 2014). Again, further regulation is achieved by mutations of *ABCC4* and *ABCG2*, in 6.2 and 5.7% of patients, respectively (cbioportal; http://www.cbioportal.org).

**Table 4:** DHEA-S and E1-S transporters and estrogen biosynthetic enzymes in ovarian cancer.

**Gene**	**Level**	**Cell line**		**Tumor tissue**	
		**Regulation**	**Reference**	**Regulation**	**Reference**
*OATP1B1*	mRNA			↑ Serous epithelial adenocarcinoma/control tissue	Svoboda et al., 2011
*OATP1B3*	mRNA	IGROV1, OVCAR-3, OVCAR-4, OVCAR5, OVCAR-8, SK-OV-3, Expressed	Lancaster et al., 2013	Cancer/control tissue ↑	Lancaster et al., 2013
		OVCAR-3, SK-OV-3, Expressed, ↑ OVCAR-3/SK-OV-3	Svoboda et al., 2011	↑ Serous epithelial adenocarcinoma/control tissue	Svoboda et al., 2011
*OATP2B1*	mRNA			↑ Serous epithelial adenocarcinoma/control tissue	Svoboda et al., 2011
*OATP4A1*	mRNA	↑YDOV-151 (mucinous adenocarcinoma), SK-OV-3/HOSE	Cho et al., 2009	≈ Serous epithelial adenocarcinoma/control tissue	Svoboda et al., 2011
		↑YDOV-139 (serous carcinoma)	Chay et al., 2010		
*OATP4C1*	mRNA			≈ Serous epithelial adenocarcinoma/control tissue	Svoboda et al., 2011
*ABCC1*	mRNA			Expressed in serous, mucinous, clear cell, endometrioid, undifferentiated cancer	Reviewed by (Ween et al., 2015)
	Protein			Positive IHC in 22–68% of ovarian cancers	Reviewed by (Ween et al., 2015)
*ABCC4*	Protein			127 OC patients; association with shorter progress free survival	Bagnoli et al., 2013
*ABCG2*	Protein	Induced in PA-1 OC cell line transfected with ESR1	Ee et al., 2004	Expressed in OC, a marker of of OC stem cells	Zhang et al., 2008
				↑ resistant OC (carboplatin + paclitaxel)	Ween et al., 2015
*STS*	mRNA			≈ EOC (9–10)/OSE (17)	Ren et al., 2015
	Protein			70% clear cell (32/45); 33% serous (6/18); and 50% mucinous adenocarcinoma (4/8)	Okuda et al., 2001
				Expressed EOC and OSE	Ren et al., 2015
				17% Cancer low/moderate, Normal ovary low (stromal cells)	Human Protein Atlas
	Activity E1-S	↑ SKOV-3, PEO-1 vs. OSE	Ren et al., 2015	↑ STS ↓ progression-free survival, epithelial OC (48), serous OC (34)	Chura et al., 2009a,b
		OC-117	Milewich and Porter, 1987		
*SULT1E1*	mRNA			↓ EOC (9–10)/OSE (17)	Ren et al., 2015
	Protein	Expressed EOC and OSE	Ren et al., 2015	17% Cancer moderate/normal ND	Human Protein Atlas
				Expressed	Ren et al., 2015
	Activity E1	↑ SKOV-3, PEO-1 vs. OSE	Ren et al., 2015		
*HSD17B1*	Activity E1 → E2	↑ SKOV-3, PEO-1 vs. OSE	Ren et al., 2015		
*HSD17B2*	mRNA			↓ EOC (9–10)/OSE (17)	Ren et al., 2015
	Protein			Expressed	Ren et al., 2015
	Activity E2			Epithelial OC (48), serous OC (34)	Chura et al., 2009b
*AKR1C3*	mRNA			↓ EOC (9–10)/OSE (17)	Ren et al., 2015
	Protein			Expressed	Ren et al., 2015
	Activity T			Epithelial OC (48), serous OC (34)	Chura et al., 2009b
*HSD3B1/2*	Activity DHEA			Epithelial OC (48), serous OC (34)	Chura et al., 2009b

*EOC, epithelial ovarian carcinoma; OSE, ovarian surface epithelia; OCCA, ovarian clear-cell adenocarcinoma, IGROV1, OVCAR-3, OVCAR-4, OVCAR5, OVCAR-8, SK-OV-3, AC-258, OC-117, PEO-1; ovarian cancer cell lines*.

The published data reveal that in ovarian cancer, E1-S and DHEA-S can enter cancer cells via higher levels of OATP1B3, and also other OATPs might contribute, such as OATP1B1 (Table 2). Uptake of these steroids might be further regulated by *SLCO* gene amplification (cbioportal; http://www.cbioportal.org). This uptake is opposed by the excretion that is catalyzed by ABCC1, ABCC4, and ABCG2, where ABCC1 and ABCG2 have the highest affinities for E1-S and DHEA-S, respectively. Amplification of genes such as *ABCC1* and *ABCG2* further modulate the excretion (cbioportal; http://www.cbioportal.org).

### Altered formation of androgens and estrogens

In endometrial and ovarian cancers, several genes that encode the enzymes for local androgen and estrogen formation from precursor steroid conjugates are expressed, and some are differentially regulated (Tables 3, 4).

Expression of the individual estrogen and androgen biosynthetic genes in endometrial cancer has been studied by several groups (for review, see Rižner, 2013). Expression of *STS*, which is required for the hydrolysis of E1-S and DHEA-S, was seen to be high and unchanged (Smuc and Rizner, 2009) or increased (Lépine et al., 2010) in cancer tissues compared to control endometrial tissue. SULT1E1 opposes the action of STS. However, the expression data for *SULT1E1* mRNA are controversial, with low and increased (Lépine et al., 2010) or unchanged (Hevir et al., 2011) expression reported for cancer tissue, as compared to control endometrium. In model cell lines for well (Ishikawa cells), moderately (RL-95 cells), and poorly (HEC-1A, HEC-1B cells) differentiated endometrial cancer (see Supplementary Table 1) sulfatase activity was detected in whole cells and in cell homogenates (Fournier and Poirier, 2009). There was no difference in the expression of *SULT1E1* between the HIEEC control endometrial cell line and the HEC-1A endometrial cancer cell line (Supplementary Table 1) (Hevir-Kene and Rižner, 2015). Among the enzymes that catalyze the activation of E1 to E2 (Figure 1), the mRNA levels of *HSD17B1* were low (Smuc et al., 2006; Lépine et al., 2010; Cornel et al., 2012), decreased (Smuc and Rizner, 2009; Lépine et al., 2010), or increased in ERα-positive G1 cancers (Cornel et al., 2012). The expression of *HSD17B7* was unchanged (Lépine et al., 2010; Cornel et al., 2012) or decreased (Smuc and Rizner, 2009), and the expression of *HSD17B12* was unchanged (Smuc and Rizner, 2009; Cornel et al., 2012) or increased (Lépine et al., 2010), in endometrial cancer. In model cell lines, the *HSD17B1, HSD17B7*, and *HSD17B12* genes were up-regulated in HEC-1A vs. HIEEC cells (Hevir-Kene and Rižner, 2015).

HSD17B2 catalyzes the oxidation of E2 back to the less potent E1, and expression of the corresponding gene *HSD17B2* was increased in endometrial cancer (Lépine et al., 2010; Cornel et al., 2012; Sinreih et al., 2013), while there was no difference in expression of *HSD17B2* among control and cancer cell lines (Hevir-Kene and Rižner, 2015). The *CYP19A1* gene encodes aromatase and is responsible for the biosynthesis of E2 *via* androstenedione or testosterone; this was weakly expressed in cancer tissue, with no significant differences seen between cancer and control endometrium (Pathirage et al., 2006; Smuc and Rizner, 2009; Lépine et al., 2010; Cornel et al., 2012). In the HHUA and HOUA model cell lines, *CYP19A1* was not expressed (Supplementary Table 1) (Sugawara et al., 2004), and there was low expression in HIEEC, Ishikawa, and HEC-1A cells (Hevir-Kene and Rižner, 2015). In tissue samples, several reports showed immunohistochemical staining for CYP19A1 in paraffin sections of endometrial cancer, where both stromal and epithelial cells were stained, and several reports have shown a lack of specific staining (for review, see Rižner, 2013). Together with our current experimental data (Sinreih et al., in review to Frontiers in Pharmacology Research Topic), it appears that in endometrial cancer the sulfatase pathway has a more important role in E2 formation.

Among enzymes that catalyze local androgen formation from DHEA-S, in addition to STS, genes encoding *HSD3B1*, and *HSD3B2* showed no differences in expression between endometrial cancer and control tissue (Sinreih et al., 2013) (Figure 1). Also in the HHUA, HOUA, Ishikawa, HEC-1A, and HIEEC model cell lines (Supplementary Table 1), these two genes *HSD3B1* and *HSD3B2* were expressed, with higher mRNA levels in the HEC-1A cells as compared to the control HIEEC cells (Sugawara et al., 2004; Hevir-Kene and Rižner, 2015). Additionally, the gene that encodes AKR1C3, which catalyzes the activation of androstenedione to testosterone, was expressed in cancer tissue, but with no significant difference seen between cancer and adjacent control tissue, although with particularly increased levels in individual patients (Rizner et al., 2006). There was also a trend for increased expression of *AKR1C3* in G2/G3 cancers compared to G1 cancers (Cornel et al., 2012), but there were also decreased levels of AKR1C3 reported in cancer compared to control endometrium (Zakharov et al., 2010). Interestingly, *AKR1C3* was up-regulated in Ishikawa and HEC-1A cells vs. the control HIEEC cells (Hevir-Kene and Rižner, 2015). These data thus supports the capacity of cancerous endometrium for formation of androgens from DHEA and DHEA-S.

In ovarian cancer, *STS* is expressed at the mRNA and protein levels in primary cell cultures, tissue samples, and model cell lines (see Supplementary Table 2) (Ren et al., 2015; Okuda et al., 2001), with no significant differences seen between ovarian carcinoma and ovarian surface epithelial cells. Using immunohistochemistry, STS was detected in 70% of clear cells, and in 33% of serous and 50% of mucinous tumors (Okuda et al., 2001). High STS activity was detected in epithelial ovarian cancer tissue (Chura et al., 2009a), and also in the SKOV-3 and PEO-1 ovarian cancer cell lines (Supplementary Table 2). STS activity was higher in the model cell lines than in control ovarian surface epithelia cells (Ren et al., 2015). Significantly decreased expression of *SULT1E1* was seen at the mRNA level in epithelial ovarian cancer cells compared to ovarian surface epithelial cells (Ren et al., 2015).

In addition to STS activity, other enzymatic activities that are also necessary for local formation of androgens and estrogen have been observed in tissue samples of ovarian cancer, including conversion of DHEA to androstenedione, and conversions between E2, E1, testosterone, and androstenedione (Chura et al., 2009b). The enzymes that catalyze these reactions (i.e., HSD17B2, AKR1C3, SULT1E1) were detected by immunohistochemistry in ovarian cancer tissue samples (Ren et al., 2015). The importance of the local formation of E2 *via* the sulfatase pathway is supported by high levels of STS and significantly down-regulated *SULT1E1* together with metabolism of E1-S to E1 (Milewich and Porter, 1987; Chura et al., 2009a) and E2 (Ren et al., 2015) in tissue samples and model cell lines of ovarian cancer. In ovarian cancer samples, androstenedione can be formed from DHEA (Chura et al., 2009a), although Ren et al. (Ren et al., 2015) found no evidence for further formation of estrogens via the aromatase pathway or activation of androstenedione to testosterone and 5α-dihydrotestosterone, as also supported by down-regulation of *AKR1C3*. However, in other studies, CYP19A1 was expressed in stroma cells of ovarian cancers (Manna et al., 2016), and has been considered a target for an endocrine therapy (Langdon et al., 2017).

## Pharmacological interventions that target intracrine actions

Endometrial and ovarian cancers are considered to be hormone-dependent cancers. As they develop mainly in postmenopausal women, they depend on local formation of active estrogens, while the roles of androgens are currently not fully understood. After menopause, the intracrine production of estrogens from the steroid precursors E1-S and DHEA-S in cancer cells can theoretically be blocked in a number of ways, including: (i) prevention of transporter-mediated uptake of E1-S and DHEA-S from the circulation; (ii) inhibition of enzymes in the intracrine pathway for the formation of active estrogens by the so-called selective intracrine modulators; (iii) prevention of estrogen actions via ERα; and (iv) induction of estrogen inactivation and excretion from cancer cells via the ABC-efflux pumps, which will decrease their intracellular concentrations. Strategies for targeting transporters, receptors and enzymes in intracrine pathways in endometrial and ovarian cancers are discussed in more detail in the following sections, with comparisons also from several studies in breast cancer.

### Sulfatase and 17-ketosteroid reductase type 1 as novel drug targets

Previous studies have suggested that in the endometrium and ovary of postmenopausal women, hydrolysis of sulfated precursors is the major pathway for the generation of active estrogens. Indeed, STS inhibitors have been successfully tested in preclinical and animal models of hormone-dependent cancers (for an extensive review on E1 sulfatase inhibitors and their efficacy in animal and human tumor models, see Thomas and Potter, 2015; Rižner, 2016). Irosustat (STX64) is a potent tricylic coumarin-based sulfamate that irreversibly blocks STS activity, and it has been examined in phase II clinical trials for the treatment of patients with advanced hormone-dependent breast and endometrial cancers (Stanway et al., 2007). Although the breast cancer data have not yet been published (ClinicalTrials.gov Identifier: NCT01662726) https://clinicaltrials.gov/ct2/show/NCT01662726), the effects in endometrial cancer were not as good as the effects of medroxyprogesterone acetate, which is in clinical use for cases of advanced/ recurrent cancer (Pautier et al., 2012).

Estrone formed by the sulfatase pathway has to be activated by reductive HSD17B enzymes, where HSD17B1 has the highest catalytic efficiency (Rižner, 2013). The inhibitors of reductive HSD17B1 enzymes can block the conversion of E1 to E2, and have potential for application to hormone-sensitive cancer therapy. Although the mRNA and protein levels of HSD17B1 are very low, this approach might be interesting for treatment of endometrial cancer, as recent reports have shown correlations between increased *HSD17B1* mRNA levels and poor prognosis (Cornel et al., 2017). This approach might also be useful in ovarian cancer, as increased reduction of E1 to E2 has been seen in ovarian cancer cells (Ren et al., 2015). Indeed, compounds have been developed that show promising inhibition of E2 formation from E1 *in vitro* (Brozic et al., 2008; Mazumdar et al., 2009), although none of these have been studied in clinical trials to date. Most likely they will not be highly effective as mono-therapeutic agents, because they prevent the conversion of a weak estrogenic E1 to the most potent estrogen E2 only, but they might be useful in combination with other inhibitors, such as inhibitors of STS.

Even if the production of E2 via the sulfatase pathway exceeds that of the aromatase pathway, blocking one enzyme might up-regulate the others. Such a mechanism was recently reported for ERα-positive breast cancer cell lines, where resistance to aromatase inhibitors was associated with up-regulation of *STS* and E1-S transporting *OATP*s (Higuchi et al., 2016). Therefore, as a consequence of inhibition of STS, the low expression levels of *CYP19A1* in endometrial and ovarian cancers (Manna et al., 2016) might be up-regulated as well. In this case, a combination of inhibitors for STS and CYP19A1 might be useful. After the introduction in around 1980 of aminoglutethimide as the first aromatase inhibitor with documented anti-cancer efficacy (Samojlik et al., 1980), the third-generation aromatase inhibitors letrozole and anastrozole, and exemestane, are currently used for treatment of postmenopausal breast cancer (Smith and Dowsett, 2003). However, in endometrial cancer, aromatase inhibitors have shown only weak effects (Bogliolo et al., 2016). For recurrent ovarian cancer their application has been suggested as well and remarkably positive effects were reported for a patient with endometrioid ovarian cancer (Pan and Kao, 2010). Dual CYP19A1-STS inhibitors have also already been designed, where the sulfatase inhibitory pharmacophore was integrated into an established aromatase inhibitor (sulfamate derivatives of letrozole and anastrozole), or *vice versa* (Woo et al., 2010; Harrelson and Lee, 2016). In a similar manner, dual HSD17B1/ STS inhibitors also represent a plausible approach in future drug discovery.

For further drug development, compounds from natural sources with dual aromatase and sulfatase inhibitory activities might also be of interest, such as a traditional Chinese herbal formula (Shu-Gan-Liang-Xue decoction) that is used for treatment of patients with breast cancer in traditional Chinese medicine (Zhou et al., 2014).

### Estrogen receptor α as a drug target

The activation of ERα by estrogens can be prevented by antagonists or selective estrogen receptor modulators (SERMs). There is an important difference between these compounds, as antagonists block estrogen action in all tissues, while SERMs can act as agonists in certain tissues, and as antagonists in other tissues, where their actions depend on the availability of co-activators and co-repressors (Traboulsi et al., 2017). The best studied SERM is tamoxifen, which has been successfully applied in the treatment of hormone-receptor-positive breast cancer for more than 40 years (see Scharl and Salterberg, 2016). In endometrium, tamoxifen has estrogenic effects and stimulates cell proliferation, which leads to hyperplasia, and eventually, to endometrial cancer (Ellis et al., 2015). Only recently has concern arisen that tamoxifen might also increase the risk of ovarian cancer, as it promotes lesions in the fallopian tubes and ovaries (Chene et al., 2014). Novel SERMs have been developed to combat estrogen-dependent cancers, including toremifene and raloxifene, together with the selective ER down-regulators, such as selective receptor downregulator fulvestrant (for reviews, see Bogliolo et al., 2017; Traboulsi et al., 2017). Further clinical studies on patients with endometrial and ovarian cancer are currently awaited.

### OAT, OATP, and ABC-transporters as novel drug targets?

The E1-S– and DHEA-S–transporting OATPs are up-regulated in endometrial and ovarian cancers. To prevent OATP-mediated uptake of steroid hormone precursors into tumor cells, blocking OATP transport would be required. Inhibition of OATP-mediated uptake has been studied extensively using synthetic and natural inhibitors of OATP1B3 and OATP1B1 to block the uptake of statins and other substrates of these OATPs, both *in vitro* and in animal studies. However, blocking the function of these two liver-specific OATPs can change the hepatic clearance of drugs, which can result in their altered pharmacokinetics (i.e., elevated plasma levels of drugs are usually expected). This may cause serious adverse reactions, such as statin-induced myotoxicity, as has been demonstrated by co-administration of cyclosporine-A and gemfibrozil with statins (for review, see Kalliokoski and Niemi, 2009; Maeda, 2015).

Moreover, studies in breast cancer have revealed that prevention of the expression of a single *OATP* is not sufficient to inhibit steroid hormone uptake (Higuchi et al., 2016). Therefore, simultaneous blocking of various OATPs would be required. However, this would also influence the physiological functions of OATPs, and would disturb the metabolic homeostasis and protection against toxins. Therefore, the clinical application of such a simultaneous block is questionable (Stieger and Hagenbuch, 2014). Furthermore, in general, this block would only work with the particular isoforms of the transporters that are restricted to the cancerous tissues (Thakkar et al., 2015). To date, only one cancer-specific isoform (cancer-type OATP1B3) has been identified, although only in colon and pancreatic cancers (Furihata et al., 2015). As the *SLCO* and *SLC* genes are amplified in a subset of patients with ovarian cancer, their targeted inhibition might be considered. Also mutations in *SLCO* and *SLC* in endometrial cancer patients might allow the development of mutation-specific inhibitors.

However, the evaluation of OATP and OAT transporters as drug targets is currently precluded by the lack of complete information about their biological functions, substrate specificities, and mechanisms of action, and the importance of the corresponding *SLCO* and *SLC* gene amplifications and mutations in patients with endometrial and ovarian cancers.

Another option would theoretically be to limit the concentrations of steroid precursors in tumor cells by increasing steroid hormone efflux via ABC-transporters. Many ABC-proteins are expressed at physiological barriers where they protect cells and tissues against toxic compounds, which include anticancer drugs (Chen et al., 2016). Some of these ABC proteins are associated with drug resistance in ovarian and endometrial cancers. For example, overexpression of ABCC1 and ABCG2 in serous ovarian cancer reduces the cellular accumulation of anticancer drugs, and this leads to the development of MDR and poor prognosis (Kunicka and Soucek, 2014; Elsnerova et al., 2016). Furthermore, ABCC1 is associated with higher tumor grade, and ABCC4 with reduced progression-free survival of patients with ovarian cancer (Ween et al., 2015). Moreover, in endometrial cancer, *ABCG2* is expressed in the Ishikawa model cell line, which is enriched in CD133 and has cancer stem cell characteristics (Nakamura et al., 2010). Inhibitors of ABC-transporters have thus been included in several clinical studies that have targeted mainly ABCB1, and also ABCC1 and ABCG2 (for extensive review, see Ween et al., 2015). However, these have not shown sufficient efficacy to block ovarian cancer progression. Higher expression of these *ABC*s should also lead to increased extrusion of steroid hormone conjugates from the cancer cells. However, in terms of the usually negative correlation between the expression levels of these ABC-transporters and patient prognosis (see above), the benefit of targeting these ABC-transporters with concurrent decreased hormone levels will not counterweigh the MDR induced.

### Current status and future prospects

In patients with advanced endometrial and ovarian cancers, targeting ERα or enzymes for estrogen activation has not proven to be successful to date (Secky et al., 2013; Mueller et al., 2015). As a small subgroup of patients (~20%) still responds to estrogen-deprivation therapy, better stratification of patients for menopausal status, hormone receptor status, and presence of estrogen biosynthetic enzymes, among other factors, could select a subpopulation that would most likely benefit from estrogen-deprivation therapy. The development of resistance to estrogen-deprivation therapy may be of major concern, and therefore a combination of drugs instead of monotherapy with one agent may extend the period of sensitivity to these therapeutics. In this respect, dual STS/CYP191 inhibitors might be important, as drugs also targeting ERα (ERα antagonists) might be given together with STS and/or HSD17B1 inhibitors. Very promising data have been reported for breast cancer, where a combination of aromatase inhibitors and novel agents that target overexpressed kinases has led to enhanced therapeutic responses (Zhao and Ramaswamy, 2014; Daldorff et al., 2017). Elucidation of such novel approaches for endometrial and ovarian cancer-specific pathways in combination with the use of selective intracrine modulators or selective estrogen-receptor modulators may lead to the development of novel therapeutic approaches to improve the success of cancer chemotherapy.

## Author contributions

TLR contributed to conception and design of the review, TLR, TT, and CÖL contributed to the final version of the manuscript, and all authors read and approved the final manuscript.

### Conflict of interest statement

The authors declare that the research was conducted in the absence of any commercial or financial relationships that could be construed as a potential conflict of interest.
