# Supplementary material for: The Importance of Steroid Uptake and Intracrine Action in Endometrial and Ovarian Cancers
Source: Front Pharmacol. 2017 Jun 19;8:346. doi: 10.3389/fphar.2017.00346 (PMC5474471; doi:10.3389/fphar.2017.00346)
Supplement: Supplementary file 1 [file SupplementaryTables1and2.docx]

**Supplementary Table 1.** Characterization of endometrial cancer cell lines.

| **Cell Line** | **Origin** | **Differentiation** | **Estrogen receptors** | **Progesterone Receptors** |
| --- | --- | --- | --- | --- |
|  |  |  |  |  |
| HEC1A | Adenocarcinoma | G2 | ERα, ERβ | PRA, PRB |
| HEC1B | Adenocarcinoma | G2 | ERα, ERβ | negative |
| HHUA | Adenocarcinoma | G1 | ERα, ERβ | PRA, PRB |
| HOUA | Adenocarcinoma | undifferentiated | negative | negative |
| Ishikawa | Adenocarcinoma | G1 | ERα, ERβ | PR |
| RL95 | Adenosquamous carcinoma | G2 | ERα, ERβ | PRA, PRB |
| AC258 | Adenocarcinoma | G3 | negative | negative |
|  |  |  |  |  |

**Supplementary Table 2.** Characterization of ovarian cancer cell lines.

| **Cell Line** | **Origin** | **Characterization** | **Mutations*** | **Estrogen Receptors** |
| --- | --- | --- | --- | --- |
|  |  |  |  |  |
| OVCAR-3 | sEOC | HGSC | *TP53* | ERα/ERß |
| OVCAR-4 | AC | HGSC | *TP53* | ERα |
| OVCAR-5 | AC | HGSC unlikely | *TP53* wild type | ER n.d. |
| OVCAR-8 | AC | HGSC possibly | *TP53, KRAS, ERBB2* | ERα, |
| SKOV-3 | AC | HGSC unlikely | *TP53*,*KRAS* Ampl. *ARIDA1A* | ERα, ERß |
| A2780 | AC | HGSC unlikely | *TP53* wild type | ERα, ERß |
| IGROV-1 | EC/CCC | mixed | *TP53,KRAS,MYC,ARIDA1A* (hypermutated) | no ERs |
| OC-117 | CCC | n.d. | n.d. | no ERs |
| YDOV-151 | MC | n.d. | wild type *TP53, BRCAII* | n.d. |
| YDOV-139 | AC | n.d | n.d. | n.d. |
| PEO-1 | sEOC | n.d. | wild type *TP53, BRCAII* deficient | ERα |
| PA-1 | teratocarcinoma | n.d. | n.d. | no ERs |

sEOC, serous epithelial ovarian cancer; AC, adenocarcinoma; EC, endometrial cancer; CCC, clear cell carcinoma; MC: mucinous carcinoma; n.d., not determined; Ampl., amplification

*mutations in TP53: criteria for high-grade serous ovarian cancer (HGSC)
